# Optimizing Home Visit Records as a Way of Improving Quality of Care: A Quality Improvement Study

**DOI:** 10.7759/cureus.75319

**Published:** 2024-12-08

**Authors:** Margarida S Neto, Catarina S Alves, Sónia Cardoso

**Affiliations:** 1 Family Medicine, Unidade de Saúde Familiar (USF) Vil'Alva, Unidade Local de Saúde do Médio Ave, Santo Tirso, PRT

**Keywords:** clinical records, domiciliary care, home visits, patient safety, primary care, quality improvement, quality of care

## Abstract

Introduction

Home visits are a key component of primary care in Portugal, designed for patients unable to visit medical facilities. However, logistical constraints often lead to incomplete real-time clinical records, impacting care quality and safety. This study aimed to improve the quality of home visit records through structural interventions and a continuous quality improvement approach.

Methods

This study was conducted in a Portuguese family health unit between February and December 2023. This retrospective study involved all home visits performed by physicians from October 2022 to October 2023. Using the Plan-Do-Study-Act (PDSA) methodology, records were assessed based on four parameters: accuracy of the “Assessment” section of the Subjective, Objective, Assessment, and Plan (SOAP) note (aligned with the International Classification of Primary Care, 2nd edition); Barthel scale documentation; updated list of problems; and updated list of chronic medication. Data were collected, analyzed descriptively, and presented at three time points: baseline evaluation (February 2023), intermediate evaluation (July 2023), and post-intervention evaluation (December 2023). Two interventions were made, including educational sessions and the introduction of physical support tools for record-keeping. The established quality-defining goal was to achieve compliance with all four parameters in at least 80% of records.

Results

At baseline, none of the 97 evaluated records met all criteria. After two interventions, compliance significantly improved. By the final evaluation, 74.7% of 95 records met all criteria, while no records failed entirely.

Discussion

Despite not fully achieving the predefined goal, interventions significantly enhanced record quality, ranging from 0% to 74.7% at the end of the study. These findings demonstrate the value of structural interventions and collaborative team efforts in improving home visit records. Despite significant progress in improving home visit records, there is still room for improvement. It is essential for healthcare professionals to continue enhancing record-keeping practices to improve the effectiveness of domiciliary care and patient outcomes.

Conclusion

This study highlights the importance of accurate clinical records for safe and effective domiciliary care. Continued commitment to structured record-keeping practices and further research is essential to sustain improvements and optimize patient outcomes.

## Introduction

Home visits are one of the primary care consultation modalities provided in Portugal. Typically, they are conducted to address the temporary or permanent inability of patients to visit their primary care physician. This form of consultation, conducted outside the family health unit, often faces logistical challenges, such as the absence of technology to facilitate real-time clinical records. Consequently, clinical records from these visits are frequently incomplete, undermining their utility for safe and effective care.

Ensuring precise records is essential for delivering high-quality care [1]. Within the complex framework of domiciliary services, thorough record-keeping is more than a procedural necessity - it forms the foundation of effective care delivery. Detailed records provide healthcare professionals with a complete overview of a patient’s medical history, individual preferences, and specific needs, enabling the provision of tailored care and promoting overall health and well-being [1]. Moreover, they enable tracking the progression or regression of a patient's health, acting as a timeline to identify patterns, anticipate potential issues, and adapt care plans accordingly [1].

In Portugal, despite the significance of home visits, the lack of informatics support during the time of the visit persistently leads to gaps in clinical records. Accurate and complete records are vital for ensuring high standards of care and effective communication among healthcare providers. They also support research and continuous quality improvement initiatives [1,2].

Efficient medical records are a fundamental element of quality improvement. Furthermore, comprehensive records support the integration of home care into broader healthcare systems, as highlighted in reviews of intensive primary care and community-based care models [3,4].

This study aims to examine and improve the quality of clinical records generated during home visits in a Portuguese family health unit. Using a continuous quality improvement approach, we seek to identify gaps in documentation practices and implement structural interventions to standardize and optimize record keeping. By doing so, we aim to enhance the quality of domiciliary care, minimize misdiagnosis and mistreatment, foster better health outcomes for patients receiving care at home, and establish a robust framework for personalized and effective service delivery.

This article was previously presented as a meeting abstract at the 41º Encontro Nacional da Associação Portuguesa de Medicina Geral e Familiar on April 5, 2024.

## Materials and methods

This study was conducted in a Portuguese family health unit between February and December 2023, serving a population of 13033 patients. The unit was composed of eight family doctors and four family medicine residents. The primary objective was to enhance the quality of records for home visits, aiming to improve the overall standard of care provided. The study adhered to the Standards for Quality Improvement Reporting Excellence (SQUIRE 2.0) guidelines and employed the Plan-Do-Study-Act (PDSA) methodology [5,6].

As a retrospective study, the population included all home visits conducted by physicians during the periods under evaluation, spanning from October 2022 to October 2023. No exclusion criteria were applied, as all visits within the specified timeframe were considered. Initially, data on home visits performed between October and December 2022 were extracted from *Módulo de Informação e Monitorização das Unidades Funcionais* (MIM@UF®), a platform used for information management and monitoring in family health units, resulting in 97 identified visits. Subsequently, specific data were collected using SClinico®, the electronic health record system utilized in primary care units in Portugal.

The analysis focused on four key parameters. The first was the “Assessment” section of the Subjective, Objective, Assessment, and Plan (SOAP) note, which is an acronym representing a widely used method of documentation for healthcare providers and a way for healthcare workers to document in a structured and organized way [7]. Investigators examined whether the content of the “Subjective” section was appropriately reflected in the “Assessment” section, with diagnostic coding aligned with the International Classification of Primary Care second edition (ICPC-2) standards. The second parameter evaluated whether the results of the Barthel scale were recorded in the "Objective" section of the SOAP note. The third parameter involved checking the accuracy and completeness of the problem list, ensuring that all chronic and relevant acute conditions under study were appropriately coded. Finally, the chronic medication list was reviewed using PEM®, an electronic prescription platform, to determine if the list only included medicines systematically prescribed within the previous year. The investigators considered that if a medicine in the chronic medication list was not prescribed during this timeframe should not be contemplated, meaning, the list was outdated.

Data were collected at three distinct time points, reflecting the update cycle of the MIM@UF® platform, which has a two-month delay in showing data. The baseline evaluation was conducted in February 2023 and analyzed data from home visits carried out between October and December 2022. The intermediate evaluation took place in July 2023, focusing on visits performed between March and May 2023. The post-intervention evaluation occurred in December 2023 and included data from visits conducted between August and October 2023.

Following the baseline evaluation, the first intervention was implemented in February 2023 through a clinical session targeting physicians. During this session, the baseline results were presented, emphasizing the importance of accurate records as a means to enhance the quality of care and improve patient safety by reducing diagnostic and prescription errors. A theoretical review of the defined parameters was also conducted to ensure team alignment and motivation. A team-wide goal was set using the SMART framework, aiming to achieve compliance with all four parameters in at least 80% of records, categorized as a “Very Good” quality standard. Quality standards were further defined as follows: insufficient (<50%), sufficient (50-65%), good (65-80%), very good (80-95%), and excellent (>95%).

In July 2023, three months after the initial intervention, results from the intermediate evaluation were presented in a clinical session to discuss strategies for further enhancement. Based on the results and team discussion, a second intervention was deemed necessary. This intervention included reinforcement of educational measures regarding proper recording, and additionally introduced physical support tools, such as paper templates, to facilitate the recording of data directly during home visits.

The final post-intervention evaluation occurred in December 2023, nine months after the initial intervention, and the final results were again presented in a clinic meeting with physicians.

Data collection and analysis were carried out exclusively by the investigators, with all information securely stored in a password-protected Microsoft Excel® (Microsoft Corporation, Redmond, WA) database.

Confidentiality was maintained throughout all phases of the study, and the collected data were used solely for research purposes. The study did not interfere with the regular functioning of the family health unit, nor did it incur any costs for the institution.

## Results

The baseline evaluation, conducted in February 2023 (analyzing home visits performed between October and December 2022), identified a total of 97 home visits at the family health unit. Key findings revealed that 96.9% (n = 94) of the records lacked the "Assessment" section of the SOAP note, coded according to ICPC-2, to reflect the diagnostic evaluation described in the "Subjective" section. Additionally, 74.2% (n = 72) omitted the results of the Barthel scale in the "Objective" section of the SOAP note, 84.5% (n = 82) failed to update the list of problems, and 83.5% (n = 81) did not revise the chronic medication list (Table 1). When considering compliance with all four evaluation criteria, none of the records fulfilled all the parameters. Only 2.1% (n = 2) met three criteria, 9.3% (n = 9) met two criteria, 36.1% (n = 35) met one criterion, and 52.6% (n = 51) failed to meet any criteria (Table 2).

**Table 1:** Distribution of the criteria evaluated during the baseline evaluation (February 2023), intermediate evaluation (July 2023), and final evaluation (December 2023). SOAP: Subjective, Objective, Assessment, and Plan.

Criteria evaluated	Baseline evaluation (February 2023) (n = 97)				Intermediate evaluation (July 2023) (n = 87)				Final evaluation (December 2023) (n = 95)			
	Met the criteria		Didn’t meet the criteria		Met the criteria		Didn’t meet the criteria		Met the criteria		Didn’t meet the criteria	
	n	%	n	n	n	%	n	%	n	%	n	%
“Assessment” section of the SOAP note, according to the “Subjective” section	3	3.1	94	96.9	61	70.1	26	29.9	89	93.7	6	6.3
Barthel scale reported in the “Objective” section of the SOAP note	25	25.8	72	74.2	80	92.0	7	8.0	92	96.8	3	3.2
Updated list of problems	15	15.5	82	84.5	58	66.7	29	33.3	82	86.3	13	13.7
Updated list of chronic medication	16	16.5	81	83.5	72	82.8	15	17.2	87	91.6	8	8.4

**Table 2:** Distribution of the number of fulfilled criteria during the baseline evaluation (February 2023), intermediate evaluation (July 2023), and final evaluation (December 2023).

Number of fulfilled criteria	Baseline evaluation (February 2023) (n = 97)		Intermediate evaluation (July 2023) (n = 87)		Final evaluation (December 2023) (n = 95)	
	n	%	n	%	n	%
None	51	52.6	2	2.3	0	0.0
One	35	36.1	6	6.9	0	0.0
Two	9	9.3	12	13.8	6	6.3
Three	2	2.1	27	31.0	18	18.9
All	0	0.0	40	46.0	71	74.7

Following the initial intervention, an intermediate evaluation was conducted in July 2023 (examining data from visits between March and May 2023), identifying 87 home visits. At this stage, the proportion of records missing the "Assessment" section coded according to ICPC-2 decreased to 29.9% (n = 26). Omission of Barthel scale results fell to 8% (n = 7), incomplete updates to the problem list dropped to 33.3% (n = 29), and failures to update chronic medication lists reduced to 17.2% (n = 15) (Table 1). Regarding overall compliance, 46% (n = 40) of the records met all four parameters. A further 31% (n = 27) met three criteria, 13.8% (n = 12) met two criteria, 6.9% (n = 6) met one criterion, and only 2.3% (n = 2) failed to meet any criteria (Table 2).

The final post-intervention evaluation in December 2023 (analyzing visits from August to October 2023) included 95 home visits. The results showed further improvements: 6.36% (n = 6) of records lacked the "Assessment" section coded per ICPC-2, 3.2% (n = 3) omitted Barthel scale results, 13.7% (n = 13) had incomplete problem list updates, and 8.4% (n = 8) did not update the chronic medication list (Table 1). By this stage, 74.7% (n = 71) of the records met all four evaluation criteria, 18.9% (n = 18) fulfilled three criteria, 6.3% (n = 6) met two criteria, and none of the records met only one or neither of the criteria (Table 2).

At baseline, no records complied with all four evaluation parameters. Following two interventions over nine months, the quality of record-keeping improved significantly. By the intermediate evaluation, compliance with all parameters increased to 46%, and by the final evaluation, it rose to 74.7%.

## Discussion

The baseline evaluation revealed that none of the home visits met the four analyzed criteria. However, at the intermediate evaluation, there was a significant improvement, with compliance rising from 0% to 46%. Over time, this effort resulted in a good quality standard (74.7%) by the end of the study, reflecting considerable progress in a relatively short period. This significant change, despite not meeting the initially defined quality standard target above 80%, highlights the commitment of healthcare professionals to improving the quality of home visit records. While it is challenging to compare these results to other family health units due to the limited published research on this specific subject, this study marks a noteworthy advancement in the quality of domiciliary care records and offers a valuable contribution for other family health units to consider and adopt.

The records of the diagnostic assessment in the SOAP section, according to the ICPC-2 classification, were the items with the most shortcomings. The challenges in completing this section stem from its inherently subjective nature, which led to variability in assessments among the healthcare providers involved. This is consistent with findings by Unwin & Jerant [8], who noted that domiciliary care can be highly individualized, and recording subjective elements accurately is often difficult. Furthermore, this highlights the need for clearer guidelines and standardized approaches for documenting such assessments to reduce inter-provider variation and improve records consistency.

In contrast, the SOAP records of the objective evaluation, including the Barthel scale, had the fewest errors, likely due to its more objective nature. The Barthel scale is widely used to assess the functional status of patients, and its clear, structured format makes it easier to complete accurately. This finding supports earlier studies on the value of standardized, measurable assessment tools in improving the accuracy and completeness of clinical records [3,4].

Regarding the chronic medication list, the primary issue was the failure to remove medications that were no longer in use, although current medications were accurately documented. This is a known challenge in clinical settings, where medication lists can become outdated and inaccurate over time [2]. The lack of regular updates to medication records can result in safety issues, such as prescription errors, potentially leading to adverse events and mistreatment. Aitken et al. [9] discuss the critical role of data management in healthcare systems, highlighting their ability to inform decision-making, optimize the use of medicines, and address inefficiencies such as polypharmacy mismanagement and medication errors, ultimately improving safety and reducing adverse events. This underscores the value of accurate and timely updates to medication records, which are essential for enhancing patient care and minimizing risks [9].

Similarly, the problem list was not updated due to the failure to remove coded signs or symptoms that lacked accompanying notes. This issue is consistent with findings in other studies where inaccurate or incomplete coding practices negatively impacted the quality of clinical records [1,9]. Efficient medical records should not only reflect the patient's current condition but also be accurate and reflective of past information to ensure ongoing care decisions are based on reliable data.

The intervention involving a structured support document proved to be an essential tool for improving the quality of domiciliary care medical records. As these records are typically completed after the visit, this support document allowed physicians to consolidate all relevant information in one place, ensuring they had a clear reminder to update and complete records later. This approach aligns with best practices, emphasizing the importance of organizational tools and structural changes in enhancing record-keeping practices and consequently, healthcare providing. Research indicates that structural interventions are often more effective than purely educational efforts alone in improving quality standards [5,6].

One limitation of this study is the inability to confirm whether the evaluated criteria accurately reflected the real situation during the home visits. The retrospective nature of the record-keeping, as well as potential omissions or inaccuracies in the post-visit records, may have influenced the accuracy of the data. Nonetheless, this study reinforces the importance of a structured approach in improving the quality of domiciliary care records. As Santomauro et al. [10] suggest, nurses and other healthcare providers in domiciliary settings must adopt strategies that ensure continuous and accurate documentation to provide safe and effective care.

In conclusion, while significant progress was made in improving the records of home visits, it is believed that there is still room for improvement in this area. Thus, it is recommended that healthcare professionals continue to use the implemented measures to maximize their potential in improving the quality of home visit records. Continued commitment to enhancing documentation practices will not only improve the effectiveness of domiciliary care but also contribute to the overall improvement of patient outcomes. Future efforts should focus on addressing the challenges related to subjective assessments and updating clinical records, especially in settings where technology may not be readily accessible. Continued quality improvement efforts in domiciliary care documentation are crucial for enhancing patient care and ensuring the accuracy and completeness of medical records. Therefore, further studies and initiatives are necessary to ensure sustained improvements in both clinical practices and the documentation process in domiciliary settings.

## Conclusions

The present study focused on improving healthcare practices by addressing the quality of clinical records during home visits. Through the implementation of quality improvement measures, significant progress was achieved in enhancing the data of home visits, particularly in meeting key evaluation parameters. This success reflects the collaborative efforts of medical professionals within the family health unit, emphasizing the importance of teamwork in family medicine.

The study highlights the value of implementing structured interventions, such as standardized support tools, to optimize care delivery and documentation processes. Beyond the positive impact on record keeping, this initiative underscores the importance of ongoing professional development and collective commitment to improving patient care. By fostering better record-keeping practices, this project contributes to enhancing the quality and continuity of care for homebound patients, further advancing the mission of primary healthcare services.
